# *In situ* measurements of human cough aerosol hygroscopicity

**DOI:** 10.1098/rsif.2021.0209

**Published:** 2021-05-05

**Authors:** Robert Groth, Luke T. Cravigan, Sadegh Niazi, Zoran Ristovski, Graham R. Johnson

**Affiliations:** International Laboratory for Air Quality and Health (ILAQH), School of Earth and Atmospheric Sciences, Queensland University of Technology, Brisbane, Australia

**Keywords:** hygroscopic growth, cough aerosol efflorescence, droplet physicochemistry, pathogen viability, organic volume fraction, bovine bronchoalveolar lavage

## Abstract

The airborne dynamics of respiratory droplets, and the transmission routes of pathogens embedded within them, are governed primarily by the diameter of the particles. These particles are composed of the fluid which lines the respiratory tract, and is primarily mucins and salts, which will interact with the atmosphere and evaporate to reach an equilibrium diameter. Measuring organic volume fraction (OVF) of cough aerosol has proved challenging due to large variability and low material volume produced after coughing. Here, the diametric hygroscopic growth factors (GF) of the cough aerosol produced by healthy participants were measured *in situ* using a rotating aerosol suspension chamber and a humidification tandem differential mobility analyser. Using hygroscopicity models, it was estimated that the average OVF in the evaporated cough aerosol was 0.88 ± 0.07 and the average GF at 90% relative humidity (RH) was 1.31 ± 0.03. To reach equilibrium in dry air the droplets will reduce in diameter by a factor of approximately 2.8 with an evaporation factor of 0.36 ± 0.05. Hysteresis was observed in cough aerosol at RH = ∼35% and RH = ∼65% for efflorescence and deliquescence, respectively, and may depend on the OVF. The same behaviour and GF were observed in nebulized bovine bronchoalveolar lavage fluid.

## 1. Introduction

Understanding the mechanisms governing the transmission and survival of respiratory pathogens has been of considerable importance in attempting to control the spread of airborne pathogens such as SARS-CoV-2 [1,2]. Fully characterizing the composition of expired cough aerosol will allow for the determination of expired particle equilibrium diameters and the micro-environments to which airborne pathogens are exposed during transport. The surface of the respiratory tract is coated with airway surface liquid (ASL), a mobile layer of mucus which can be aerosolized during expiration events. The composition of ASL is understood to consist primarily of water and solids, including mucins (mucin 5AC and 5B), inorganic salts (NaCl, KCl), and pulmonary surfactants [3–5]. These inorganic salts, and to a lesser extend the mucins, are hygroscopic, so that when droplets containing these species are exposed to the ambient environment, the droplets will grow or evaporate to reach a thermodynamic equilibrium with the relative humidity (RH) of the environment. During hydration from a crystalline state, some salts exhibit an abrupt transition to a liquid state (deliquescence), and through dehydration from an aqueous state may also exhibit a prompt recrystallization (efflorescence). Understanding the hygroscopicity and state hysteresis of human respiratory aerosol is an important step in determining both the composition of the micro-environment to which airborne pathogens are exposed, and the equilibrium particle sizes required to predict airborne dynamics.

There are several key challenges when investigating the physico-chemical characteristics of respiratory aerosols. The coughing process aerosolizes a very small volume of respiratory fluid (picolitres) compared to the volume of air during expiration (litres) [6,7]. The resulting low mass concentration causes difficulties in conducting compositional analyses, as cough aerosol must to be collected over an extended period of time to accumulate sufficient material for analysis [8]. The count median diameter (CMD) of these respiratory droplets has been measured to be less than 2 µm [6,7,9], which is generally too small to suspend precisely in an electrodynamic balance (EDB) for hygroscopicity measurements [10–14]. The interpersonal variability of ASL composition [15] makes it difficult to develop precise analogues for respiratory fluid, as changes in the organic concentration will affect particle hygroscopicity and therefore equilibrium diameter and airborne transport. Respiratory aerosol is produced through bubble bursting [16], which in sea spray aerosol, has been shown to result in enriched organic concentrations at small particle diameters [17–20], suggesting that the aerosol produced through coughing may differ in organic concentration in comparison to bulk ASL collected through bronchoalveolar lavage (BAL).

Pathogens transferred through cough droplets can contaminate surfaces or be spread through the air in two primary airborne transmission routes. The first is through large ‘ballistic' droplets [21] depositing directly onto mucous membranes, and the second is through the inhalation of cough aerosols or droplet nuclei (DN). A droplet nucleus is the residual particle after the primary respiratory droplet has partially evaporated [22]. The size and state (liquid or dry DN) of respiratory aerosols is dependent on ambient RH due to hygroscopic salts present within respiratory droplets. Because of the potential for complex interaction between proteins, surfactants, and hygroscopic salts within the respiratory fluid, it is not fully understood how humidity changes can affect airborne pathogen viability or the size of the droplets. Many studies have been conducted investigating the survival of airborne pathogens in pure water, saliva, and ASL analogues [23–32]; however, it has proven to be difficult to conduct *in vivo* experiments to measure pathogen viability in the cough aerosol of infected individuals.

It is clear that many factors, including airborne suspension times and pathogen viability, are dependent primarily on particle diameter, and thus, particle composition. Understanding the hygroscopicity of respiratory aerosol will provide a link between equilibrium particle diameter and particle composition.

## 2. Material and methods

### 2.1. Participant selection

The experimental study was fully scrutinized and cleared by the Queensland University of Technology Research Ethics Committee. Subjects aged between 19 and 60 years of age were recruited via an e-mail broadcast. Subjects were asked to self-exclude if they were smokers, experiencing illness, asthma sufferers, had recently experienced respiratory problems or were likely to experience discomfort in confined spaces. In total, there were 10 participants who did not self-exclude and participated in this study. Out of these 10 participants, five were excluded from hygroscopicity analysis due to insufficient aerosol production or if the measured growth factors at low RH (RH less than 40%) were consistently not close to unity.

### 2.2. Collection of bovine respiratory fluid

Bovine bronchoalveolar lavage fluid (B-BALF) was collected from the lung of a deceased cow. The cow was killed as part of routine commercial food production, and the lung was freshly recovered by the processing personnel and immediately transferred to a sterile container for lavage. The lung was washed thoroughly with distilled deionized water, and through massaging and gravity, the B-BALF was collected in a sterile bottle and sealed.

### 2.3. Aerosol collection and storage

The 400 l rotating drum described by Johnson *et al*. was used in this study to maintain aerosol suspension during the hygroscopicity tandem differential mobility analyser (H-TDMA) measurements at a rotation rate of 1.7 r.p.m. [33,34]. An H-TDMA uses a differential mobility analyser (DMA) to select a monodisperse aerosol fraction, of a precisely specified diameter, from an initial polydisperse aerosol sample. The monodisperse fraction is then humidified to a target RH before remeasuring its size using a scanning mobility particle sizer (SMPS). The participants breathed HEPA filtered room air for 2 min prior to coughing to purge ambient aerosol particles from their lungs, and then subsequently coughed at a comfortable pace and intensity into the drum for 2 min. While a participant was coughing, the internal drum pressure was maintained at ambient pressure by setting the valves to allow air to leave the drum during exhalation and to allow filtered room air to enter the drum during inhalation. The provision of HEPA filtered room air during inhalation was a necessary measure to prevent contamination of the sample by room air, as even a small leak of room air had the potential to contribute a large number of particles to the cough sample, preventing accurate analysis. The H-TDMA drew air from the drum at 0.6 l min^−1^ which was replaced by HEPA filtered room air. In a separate experiment, B-BALF was nebulized directly into the clean drum with a Collison nebulizer using filtered air at a flow rate of 1 l min^−1^, with the remaining procedure unchanged. The B-BALF mixture was composed of 2 ml B-BALF and 8 ml distilled deionized water. The initial size distribution of each participant's cough aerosol was measured using an SMPS, and the average CMD was measured to be 101.6 nm and the average geometric standard deviation (GSD) was measured to be 1.98.

### 2.4. Aerosol growth and measurement

**Figure 1.**
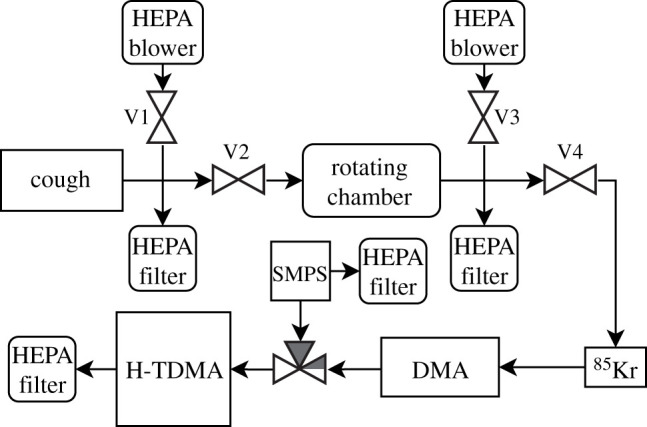
Experimental schematic of the system used to suspend respiratory aerosol and measure hygroscopic growth.

A schematic of the experimental set-up is shown in figure 1. Aerosol was drawn from the rotating chamber at 0.6 l min^−1^ through a silica gel diffusion dryer and Kr-85 aerosol charge neutralizer. The aerosol size distribution was then measured with a scanning mobility particle sizer (SMPS), using a TSI 3787 condensation particle counter (CPC) (TSI, Shoreview, MN) and a custom manufactured DMA (specifications in electronic supplementary material, table S1). The first DMA had a sheath flow rate of 4 l min^−1^ and selected a monodisperse sample at a D_dry_ of 100 nm (RH < 10%), and the resulting monodisperse aerosol was subjected alternately at 2-min intervals to either humidification (initial RH < 10%) or desiccation (initial RH > 90%) to measure both hydration and dehydration behaviour. The aerosol carrier flow out of this humidity-dependent path was then passed through a humidification sheath and conditioned to 90% RH. The H-TDMA was then used to measure the particle size distribution at 90% RH after hydration and dehydration to determine the diametric growth factor (GF), with a sheath flow rate of 3.6 l min^−1^. The RH in the carrier flow was then incrementally lowered to 10% RH over a period of approximately 2 h, with corresponding size distributions to measure the diametric growth factor as a function of RH.

## 3. Results

### 3.1. Hydration and dehydration of respiratory aerosol

**Figure 2.**
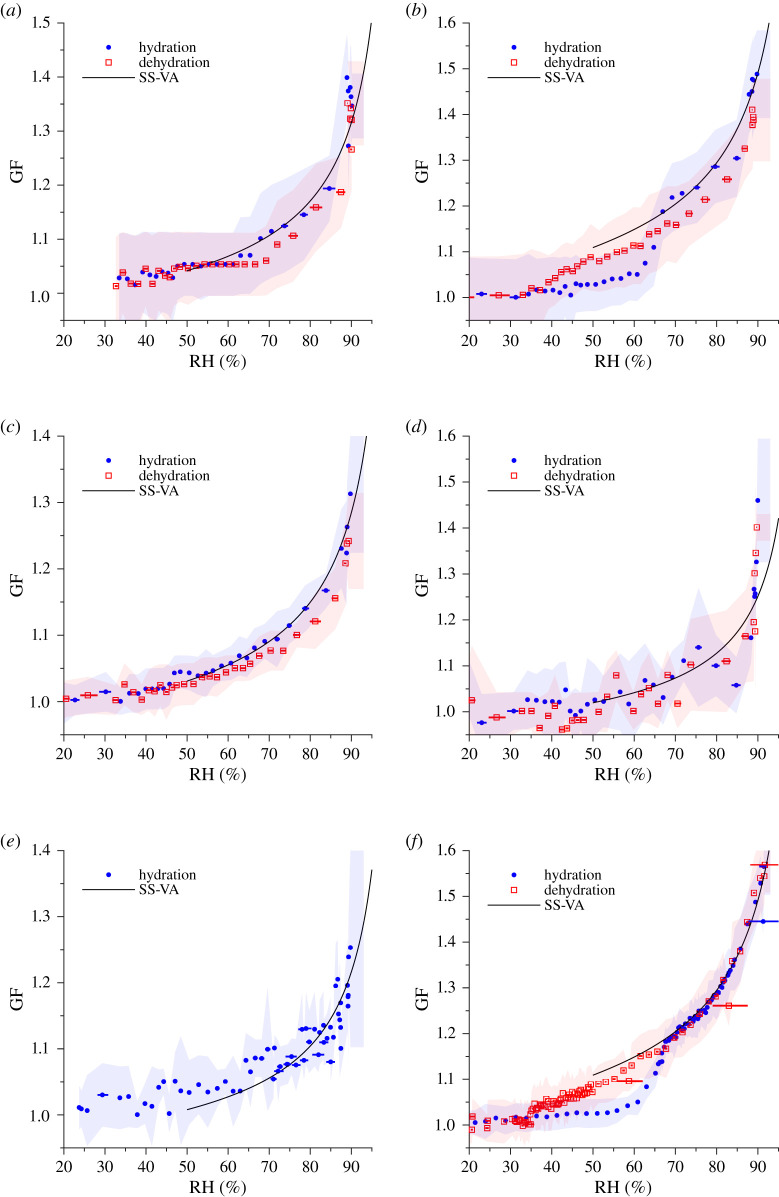
Diametric growth factor (GF) measurements of (*a*) participant A, (*b*) participant B, (*c*) participant C, (*d*) participant D, (*e*) participant E, and (*f*) bovine bronchoalveolar lavage fluid (B-BALF), at a series of relative humidity (RH) values. The best-fit separate-solute volume additivity (SS-VA) model is accompanied (black) and the standard error of the GF (shaded bands) as retrieved from the TDMAinv algorithm.

Figure 2 shows the effect of hydration and dehydration on the diameter of respiratory aerosol from the coughs of the participants and the B-BALF. The GF as a function of RH was measured using an H-TDMA, with a 2 min time interval between hydration and dehydration scans. All post humidification H-TDMA data were inverted using the TDMAinv algorithm [35].

The variability between participants, in both number concentration and composition, has shown to be common in human expiration studies [6–9,15,36–38]. Repeated hygroscopicity measurements in higher number concentration samples are typically similar (figure 2*a,b*,*c*,*f*), however low number concentration samples (electronic supplementary material, figure S2) typically show larger variability between measurements at similar RH (figure 2*d*,*e*). Having higher particle number concentrations increase the likelihood that an individual hygroscopicity measurement will be representative of the typical particle at the selected diameter. Due to the low aerosol material volume produced during expiration relative to the total volume of air expired, H-TDMA measurements of participants with low aerosol number concentrations will not be suitable for analysis.

## 4. Discussion

### 4.1. Hygroscopicity measurements

**Figure 3.**
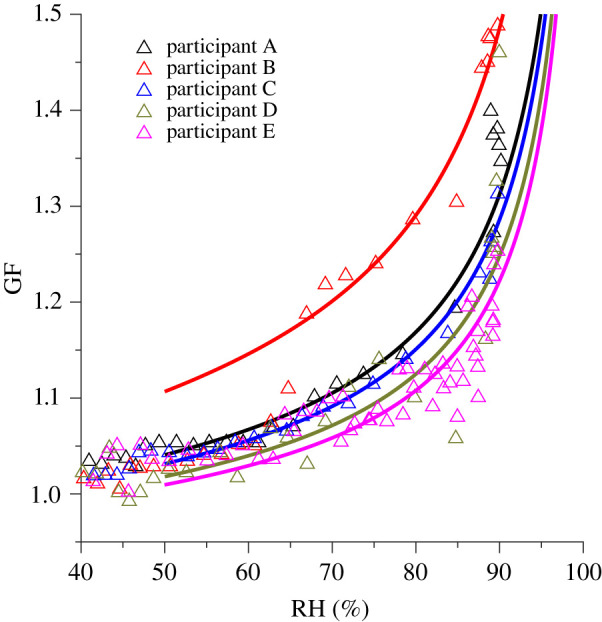
Comparison between each participant's cough aerosol diametric growth factor (GF). The best-fit separate-solute volume additivity (SS-VA, solid line) accompanies the raw data (triangles). Participant A is represented in black, participant B in red, participant C in blue, participant D in green and participant E in magenta.

The airborne dynamics of expired ASL are governed primarily by the diameter of the particles [39]. For this reason, an accurate understanding of how the particles will grow or shrink as they exchange water vapour with the atmosphere in response to changing RH is necessary to predict the transport routes of potentially pathogen-laden airborne respiratory droplets or DN. The average GF value measured at 90% RH in this study was 1.31 ± 0.03, and is lower than the values observed in artificial ASL studies, indicating a difference in composition or aerosol generation process [32,40–42]. The average diametric growth factors are presented in the electronic supplementary material (electronic supplementary material, table S5) at 40%, 60% and 90% RH. Figure 3 shows the comparison of each participant's GF measurements. It can be assumed that the cough aerosol of participants A, C, D and E are similar in composition (table 1) due to similarities in hygroscopicity; however, participant B exhibits distinctly different behaviour.

**Table 1.** Organic volume fraction estimates of the respiratory droplets of each participant using the Zdanovskii–Stokes–Robinson (ZSR) mixing rule, the compressed film model (CFM), and the separate-solute volume additivity (SS-VA) model for each participant, the B-BALF, and a known mixture of media from Niazi *et al*.

	organic volume fraction of dry solute						
	human participants					nebulized mixture	
	A	B	C	D	E	B-BALF	media [32]
ZSR	0.86 ± 0.02	0.76 ± 0.05	0.87 ± 0.01	0.89 ± 0.02	0.90 ± 0.01	0.74 ± 0.01	0.51 ± 0.02
CFM	0.88 ± 0.02	0.77 ± 0.06	0.89 ± 0.01	0.91 ± 0.02	0.93 ± 0.01	0.76 ± 0.01	0.52 ± 0.03
SS-VA	0.91 ± 0.01	0.84 ± 0.04	0.92 ± 0.01	0.93 ± 0.01	0.94 ± 0.01	0.84 ± 0.01	0.62 ± 0.02
average	0.88 ± 0.03	0.79 ± 0.04	0.89 ± 0.02	0.91 ± 0.02	0.92 ± 0.02	0.78 ± 0.05	0.55 ± 0.06

### 4.2. Organic volume fraction estimates

**Figure 4.**
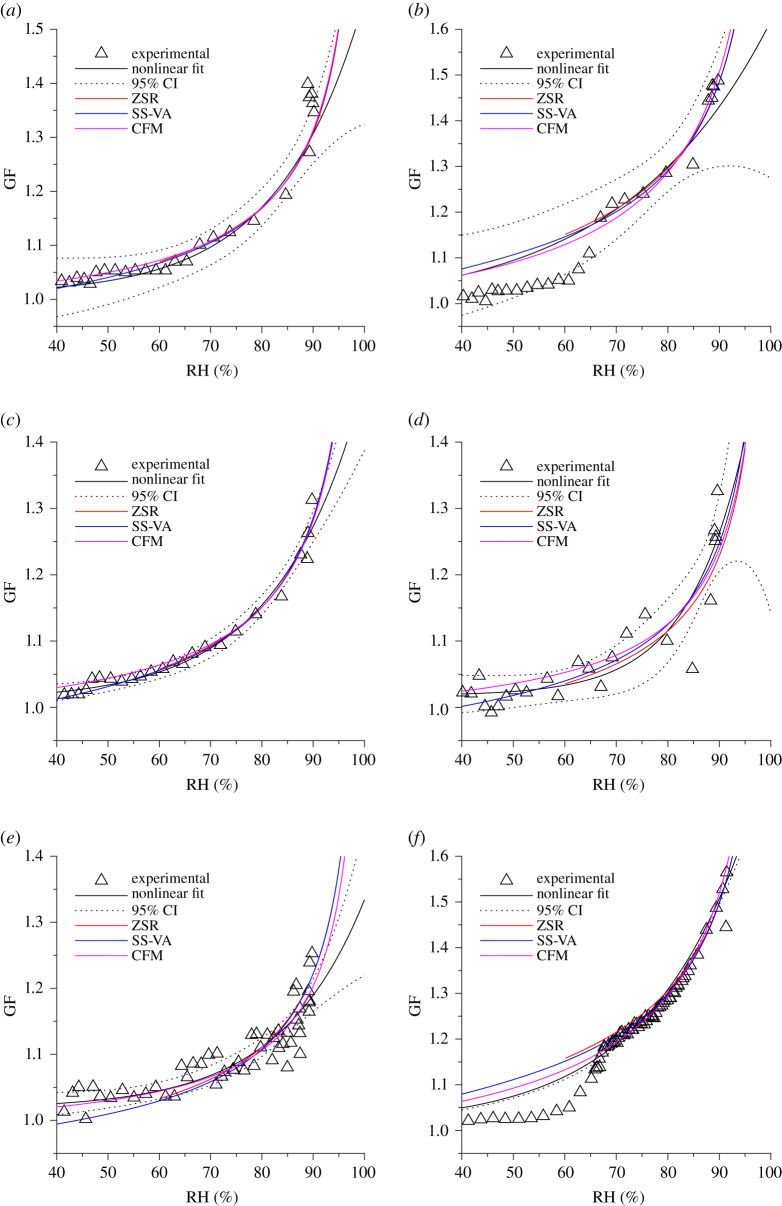
Physical models and a nonlinear model, with 95% confidence intervals, fit to the experimental hydration data. The equilibrium relative humidity (RH) for each model was calculated varying only the organic volume fraction (OVF), using least-squares fitting to determine the best OVF for (*a*) participant A, (*b*) participant B, (*c*) participant C, (*d*) participant D, (*e*) participant E and (*f*) bovine bronchoalveolar lavage fluid (B-BALF).

For each H-TDMA measurement, hygroscopicity models were implemented to estimate the OVF. To achieve this, a nonlinear model of the form GF(RH) = *β*_0_ + *β*_1_exp(*β*_2_RH) was fit to the experimental data. The data used in the fit were from the hydration scans above the identified deliquescence RH (DRH) if it existed. In this study, deliquescence was observed only in the H-TDMA data of participant B and in the B-BALF (figure 2*b*,*f*, respectively). If deliquescence was not observed, the model was fit across the range of RH. The physical models were then computed allowing the OVF to vary and then compared to the nonlinear model using least-squares fitting to determine the OVF which best represented the experimental data for each physical model. The Zdanovskii–Stokes–Robinson (ZSR) mixing rule was implemented using the osmotic pressure parameterization of protein hygroscopicity [43,44] in conjunction with Pitzer's parameterization of the osmotic coefficient of NaCl [45]. The compressed film model (CFM) was implemented using parameters typical of seawater (electronic supplementary material, table S3) to account for the surfactant effect of the organics in the droplets [20,46,47]. Finally, the separate-solute volume additivity (SS-VA) model was implemented, which accounted for the hygroscopic effects of the protein and the salt in the solution [43]. All models were implemented under the simplified solute assumption that only mucin and NaCl were present in water. Porcine gastric mucin (PGM) was chosen as the organic component (material data presented in electronic supplementary material, table S2 [48,49]) because the primary mucin in both porcine gastric mucus and human respiratory mucus is mucin 5AC [50,51], and therefore it is assumed that PGM is sufficient to represent the mucus found within ASL as a first approximation [40]. All models assume an initial dry diameter of 100 nm, which is the same as the dry monodisperse size that was selected by the initial DMA. All models were implemented using a temperature of 293.15 K. The hygroscopicity models computed with each best fit OVF are presented in figure 4 alongside the nonlinear fit and its 95% confidence intervals.

From these models, the OVF is estimated and presented in table 1. The average estimated OVF is 0.88 ± 0.07 and is in agreement with a range of estimates provided in the literature [8,40,41,52]. The same method was used using a known mixture as a comparison. In the work of Niazi *et al*., a solution was nebulized composed of 10 g NaCl, and 8 g BSA, which corresponds to a solute OVF of approximately 0.62 [32]. The SS-VA model, using the density and molecular weight of both PGM and BSA [43], is in good agreement with this OVF (electronic supplementary material, table S4). The CFM and the ZSR mixing rule, however, underestimate the OVF in this nebulized mixture. This discrepancy indicates that for respiratory aerosol below supersaturation, the ZSR rule and the CFM may not accurately predict hygroscopic growth. For experiments that aim to model the airborne dynamics or pathogen viability in respiratory aerosol, it is essential that the composition is representative of what is measured in real participants' expiratory aerosol due to interactions between the components of the droplets, which will affect the particle diameter and thus airborne dynamics and ionic concentration of the micro-environment to which the pathogens are exposed.

### 4.3. Hysteresis behaviour

**Figure 5.**
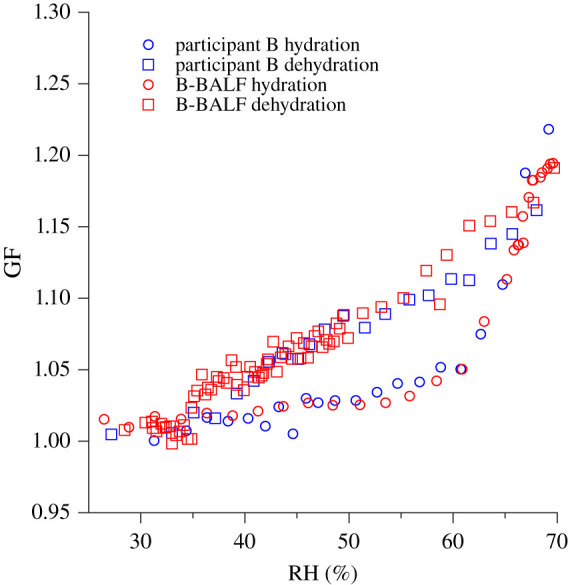
Comparison between efflorescence and deliquescence measurements of participant B (blue) and the B-BALF (red).

Figure 2*b* shows evidence of deliquescence at approximately 65% RH. The DRH of a multiple component system is expected to be lower than what is observed in a binary NaCl solution as this phenomenon has been observed previously in complex organic–inorganic solutions and mixtures, such as SSA [53,54]. The same behaviour is also observed at 65% RH in figure 2*f*, suggesting that nebulized B-BALF may be a useful analogue for the human respiratory aerosol of some humans (figure 5). The mass fraction of the non-ionic components of respiratory aerosol have previously been estimated to account for approximately 90% of the solute mass [40,52] and the bulk of this organic component is also expected to be proteinaceous. Proteins have the capacity to retain some water after dehydration and will not crystallize in these conditions. Because of proteinaceous water retention, it is expected that efflorescence would occur on a longer timescale than would be observed in a binary NaCl solution. This is consistent with the observed behaviour in the dehydration measurements shown in figure 2*b* at approximately 35% RH, where gradual efflorescence may be occurring. Once again, this is observed the B-BALF sample (figure 2*f*) where efflorescence is observed at 35% RH.

Hysteresis was observed in the cough aerosol of the participant with the lowest estimated OVF, indicating that there may exist some critical protein concentration required for hysteresis. The work of Mikhailov *et al*. shows the hygroscopic growth and hysteresis behaviour of BSA:NaCl mixtures with different mass fractions of BSA [43]. Those results showed that the efflorescence RH (ERH) was decreasing with increasing organic concentrations, and there was likely microstructural rearrangement during hydration and deliquescence. The findings of Mikhailov *et al*. indicate that between 75% and 90% organic mass fraction (OMF), corresponding to 82.6% and 93.5% OVF, respectively, the hysteresis behaviour becomes largely suppressed. Based on the OVFs estimated by the SS-VA, presented here, there may exist a critical protein concentration for which hysteresis becomes suppressed between 84% and 91% OVF. Current physical models are limited in predicting the physical behaviour of proteinaceous components of aerosol, and further work must be done to incorporate physical processes such as liquid–liquid phase separation and vitrification. Identification of systems in which efflorescence occurs is of particular importance, as the physical state of the particles will affect the viability of embedded viruses [32]. The use of B-BALF as an additional comparison is very useful here as it shows good agreement with the behaviour of a human participant. This shows potential for the use of B-BALF as an analogue for human ASL and could potentially be incorporated into airborne pathogen viability experiments to ensure the suspension fluid accurately represents the environment in which these pathogens would exist after being expired in human respiratory aerosol.

### 4.4. Droplet equilibrium diameters

An equation presented by Nicas *et al*. for calculating the size of a droplet immediately after being generated has been applied in the form of equation (4.1), to determine the droplet diameter before evaporation. The corresponding ‘evaporation factor' (EF) can also be calculated, where *C*_solute_ is the concentration of the solute in the solution, *ρ*_solute_ is the density of the solute, *d*_eq_ is the equilibrium diameter of the droplet at a given RH, and *d*_drop_ is the diameter of the droplet before any evaporation has commenced [52].

4.1 $$\begin{array}{c} d_{\mathrm{eq}}=d_{\mathrm{drop}}{\left(\frac{C_{\mathrm{solute}}}{\rho_{\mathrm{solute}}}\right)}^{1/3}=d_{\mathrm{drop}}EF. \end{array}$$

Using the OVF estimates from the SS-VA model implemented in this work, equation (4.2) was used to determine the corresponding mucin mass concentration (*C*_muc_) in the respiratory droplets, assuming a reasonable NaCl concentration of 8.8 g l^−1^ [52].

4.2 $$\begin{array}{c} C_{\mathrm{muc}}=\frac{\rho_{\mathrm{muc}}C_{\mathrm{NaCl}}\mathrm{OVF}}{\rho_{\mathrm{NaCl}}(1-\mathrm{OVF})}. \end{array}$$

Combining equations (4.1) and (4.2), and expressing the density of the total solute as the volume weighted sum of the density of both solutes, equation (4.1) can be expressed as

**Figure 6.**
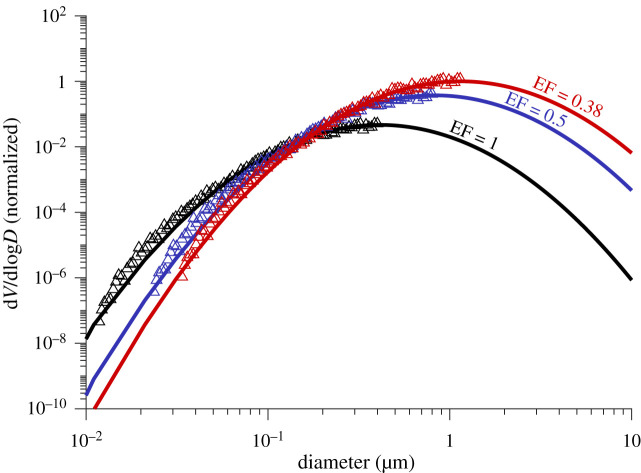
Average volume distribution for all participants' cough measurements. The data after evaporation (black, EF = 1) were measured using a scanning mobility particle sizer (SMPS). The distribution measured in this study was then corrected before any evaporation using EF = 0.5 (blue) from Nicas *et al*., and then EF = 0.38 (red, this work electronic supplementary material, table S6). The volume distributions are normalized to the maximum volume.

4.3 $$\begin{array}{c} d_{\mathrm{eq}}=d_{\mathrm{drop}}{\left(\frac{C_{\mathrm{NaCl}}}{\rho_{\mathrm{NaCl}}(1-\mathrm{OVF})}\right)}^{1/3}=d_{\mathrm{drop}}\mathrm{EF}, \end{array}$$

for respiratory droplets under the simplified solute assumption. The EF at 0%, 40%, and 60% RH for each participant is calculated and reported in the electronic supplementary material (electronic supplementary material, table S6). The average EF estimate at 0% RH is 0.36 ± 0.06 and indicates that the respiratory droplets will reduce in size by a factor of approximately 2.8 of their initial diameters when reaching an equilibrium at 0% RH, or if the droplets effloresce and recrystallize. If there is no recrystallization, then in typical comfortable room RH (40% < RH < 60%) it is expected that the droplets will reduce in size by a factor of approximately 2.6 with an EF of 0.38 ± 0.05. These predictions also estimate the concentration of proteins in evaporated droplets to be 60 ± 10 g l^−1^, similar to what was measured by Effros *et al*. [8]. The particle size distribution of each participant was measured, and the average volume median diameter (VMD) was determined to be 0.41 µm (figure 6). After applying the EF presented in this study, the VMD was increased to 1.14 µm before any evaporation, compared to 821 nm using EF = 0.5 from Nicas *et al*. [52]. The decreased initial droplet size scaling factor (EF) presented here indicates that the initial volume of respiratory droplets immediately after expiration may be larger than previously estimated, meaning that the concentration of pathogens embedded within equilibrium droplets may also be higher than previously estimated. Combining this new estimate of the EF with information about the size distribution of cough aerosol, concentration of virions within the respiratory tract, fluid dynamic models of droplet transport, and the distribution of virions within respiratory droplets, useful probabilistic models can be developed for predicting transmission potential of infected individuals [41,55].

## 5. Conclusion

The hygroscopicity of expired human respiratory droplets of several participants and B-BALF was measured using an H-TDMA. Physical models with simple assumptions of the overall composition were compared to the measured values to estimate the OVF of the aerosol, and it was found that organics account for an estimated 0.88 ± 0.07 of the dry solute volume. The initial diameter of the droplets prior to any evaporation was calculated to determine how much water is lost from the droplets in typical indoor atmospheric conditions and the resulting diameter of the droplet. The average GF of the human samples at 90% RH was measured to be 1.31 ± 0.03. Hysteresis behaviour was observed in one sample at approximately 65% and 35% RH for deliquescence and efflorescence, respectively. This behaviour was observed in the human sample with the lowest estimated OVF, indicating that the mechanisms that determine the hydration dependent state transitions of human ASL droplets may depend on the organic concentration. It was determined that bovine B-BALF can exhibit similar hygroscopic growth and hysteresis behaviour of some human cough aerosol, indicating that animal B-BALF may be a valuable substitute for human respiratory fluid in nebulization studies. The three models implemented in this work describe only water uptake of the droplets, and further work into predicting conditions under which respiratory droplets effloresce will provide critical insight into the physico-chemistry and airborne dynamics of respiratory droplets at typical room RH values, having implications for airborne pathogen viability and preventing the spread of infectious diseases. The evaporation factor of respiratory droplets in dry air was estimated to be 0.36 ± 0.05, which if combined with cough aerosol particle size distributions, can provide insight into the volume and sedimentation rates of respirable pathogen-laden aerosol generated during coughing. Further work investigating the suitability and variability of bovine bronchoalveolar fluid as an analogue for human respiratory fluid may prove very useful for investigating the viability of respiratory pathogens.

## Data accessibility

Raw participant size distribution measurements are presented in the electronic supplementary material. Processed H-TDMA data and unprocessed SMPS data can be accessed online at https://doi.org/10.5061/dryad.fn2z34tt8 [56].
